# Methyl Jasmonate and Methyl-β-Cyclodextrin Individually Boost Triterpenoid Biosynthesis in *Chlamydomonas Reinhardtii* UVM4

**DOI:** 10.3390/ph14020125

**Published:** 2021-02-05

**Authors:** Audrey S. Commault, Unnikrishnan Kuzhiumparambil, Andrei Herdean, Michele Fabris, Ana Cristina Jaramillo-Madrid, Raffaela M. Abbriano, Peter J. Ralph, Mathieu Pernice

**Affiliations:** 1 Climate Change Cluster, University of Technology Sydney, Ultimo, NSW 2007, Australia; unnikrishnan.kuzhiumparambil@uts.edu.au (U.K.); andrei.herdean@uts.edu.au (A.H.); michele.fabris@uts.edu.au (M.F.); anacristina.jaramillomadrid@alumni.uts.edu.au (A.C.J.-M.); raffaela.abbriano@uts.edu.au (R.M.A.); peter.ralph@uts.edu.au (P.J.R.); mathieu.pernice@uts.edu.au (M.P.); 2 Synthetic Biology Future Science Platform, CSIRO, Brisbane, QLD 4001, Australia

**Keywords:** triterpenes, natural product, microalgae, elicitors, squalene, sterol, white biotechnology

## Abstract

The commercialisation of valuable plant triterpenoids faces major challenges, including low abundance in natural hosts and costly downstream purification procedures. Endeavours to produce these compounds at industrial scale using microbial systems are gaining attention. Here, we report on a strategy to enrich the biomass of the biotechnologically-relevant *Chlamydomonas reinhardtii* strain UVM4 with valuable triterpenes, such as squalene and (*S*)-2,3-epoxysqualene. *C. reinhardtii* UVM4 was subjected to the elicitor compounds methyl jasmonate (MeJA) and methyl-β-cyclodextrine (MβCD) to increase triterpene yields. MeJA treatment triggered oxidative stress, arrested growth, and altered the photosynthetic activity of the cells, while increasing squalene, (*S*)-2,3-epoxysqualene, and cycloartenol contents. Applying MβCD to cultures of *C. reinhardtii* lead to the sequestration of the two main sterols (ergosterol and 7-dehydroporiferasterol) into the growth medium and the intracellular accumulation of the intermediate cycloartenol, without compromising cell growth. When MβCD was applied in combination with MeJA, it counteracted the negative effects of MeJA on cell growth and physiology, but no synergistic effect on triterpene yield was observed. Together, our findings provide strategies for the triterpene enrichment of microalgal biomass and medium.

## 1. Introduction

With more than 20,000 different molecules reported to date, triterpenes are one of the widest classes of natural products, of which the highest diversity is found in the plant kingdom [1,2]. Triterpenoids comprise structurally diverse compounds that are involved in primary or secondary metabolism. Sterols, the only triterpenes that belong to the primary metabolism [3], are key structural components of cell membranes, and act as signalling molecules (steroidal hormones). Other triterpenes are not regarded as essential for normal growth and development, but do contribute to the plant defence mechanism against abiotic and biotic stress [2]. Several triterpenes have a large range of industrial applications in the food and cosmetics sectors, as well as significant potential with pharmaceuticals [2,4]. For instance, phytosterols are used to lower blood LDL cholesterol [5], ergosterol is used for the synthesis of vitamin D_2_ (ergocalciferol) [6], and the plant triterpene betulinic acid has shown promise for the treatment of HIV and certain cancers in animal models [7,8].

Triterpenes derive from squalene, a molecule produced by the condensation of prenyl phosphates (forming farnesyl pyrophosphate, FPP) synthesised by the mevalonate pathway and/or the plastidial 2-C-methyl-D-erythritol 4-phosphate (MEP) pathway. Plants possess both pathways, while the unicellular green microalga *Chlamydomonas reinhardtii* possesses only the MEP pathway. Squalene is then converted to (*S*)-2,3-epoxysqualene (also named 2,3-oxidosqualene), the last common intermediate of sterols and other triterpenes. (*S*)-2,3-epoxysqualene can then be cyclised into cycloartenol for sterol production or other triterpenes precursors by oxidosqualene cyclases (OSCs) collectively known as triterpene synthases. Squalene in itself is a valuable molecule widely used in medicine, food, and cosmetics as a vaccine adjuvant, an antioxidant, and an anti-aging compound [9]. Squalene has been traditionally extracted from shark liver oil, but environmental concerns have motivated its extraction from other sources such as vegetable oils or fast-growing microorganisms including yeasts, bacteria, and microalgae [9]. The triterpene intermediate, (*S*)-2,3-epoxysqualene, is also an interesting molecule for production of high-value pharmaceutically-relevant triterpenoids in genetically engineered organisms [4,10,11]. 

Despite their huge pharmaceutical potential, triterpenoids are the least engineered class of terpenoids [4]. Yet the difficulties of producing and purifying large quantities of industrially-relevant triterpenes from their natural sources coupled with environmental concerns are driving the need for more sustainable production platforms. Bacteria and yeasts are often considered the most appropriate candidates for heterologous production, but photosynthetic microorganisms are emerging as alternate candidates [9]. Indeed, green microalgae naturally produce intermediates of plants triterpenoid synthesis, although the pool of intermediates need to be increased by genetic engineering to maximise the yield of the desired plant triterpene and avoid competition with the microalga metabolism. Photosynthetic microorganisms also have the advantage to require low inputs (sunlight, nutrients and CO_2_) for growth, thereby minimising the environmental impact. Additionally, the algal residual biomass generated during the extraction process can be further valorised by the production of other value-added molecules (e.g., pigments, feed, oils) following a multi-product algal bio-refinery approach [10]. In this regard, the unicellular green microalgae, *Chlamydomonas reinhardtii*, appears to be a promising candidate for triterpenoid engineering, and researchers are actively working on new strains with higher transformation efficiency. The cell-wall deficient UV mutated *C. reinhardtii* strain UVM4 was recently created to overcome low nuclear transgene expression in *C. reinhardtii* [11] and it was successfully used to produce the sesquiterpenoid patchoulol through genetic engineering [12]. We recently demonstrated that *C. reinhardtii* cells (wild-type strain 137c) responded to methyl jasmonate (MeJA) treatment (1 mM) by up-regulating the MEP pathway leading to the accumulation of the triterpenoids precursors FPP, squalene and (*S*)-2,3-epoxysqualene. MeJA treatment is therefore a useful strategy for the accumulation of triterpene precursors and intermediates in *C. reinhardtii* [13].

The current study investigated the response of strain UVM4 to two elicitors MeJA and methyl-β-cyclodextrin (MβCD) as production strategies to enrich the microalgal biomass with triterpenoid precursors such as squalene and (*S*)-2,3-epoxysqualene, and to accumulate sterols in the growth medium, thereby simplifying the extraction procedure. The supplementation of exogenous MeJA and cyclodextrins (CDs) to in vitro plant cultures has emerged as a novel approach for the hyperaccumulation of secondary metabolites and the discovery of new molecules [14,15,16]. In plants, the addition of exogenous MeJA to in vitro cultures is known to induce an oxidative stress via the production of reactive oxygen species (ROS) [14]. While we have previously described a similar stress response to MeJA in *C. reinhardtii* (wild-type strain 137c) [13], this response has been further monitored in the current study. In addition, we report the effect of the elicitor MβCD in *C. reinhardtii* strain UVM4. Cyclodextrins have amphiphilic characteristics, which allow them to form complexes with hydrophobic compounds, such as triterpenes, facilitating their export from cells and their isolation from the culture medium [17]. By stimulating extracellular accumulation of triterpenes, CDs limit potential retroinhibition processes and product toxicity in the producing cells, therefore enhancing triterpene production [17]. Although intensively used in plants, the ability of MβCD to increase terpene production has not been tested in any microalgae. We also hypothesised a synergistic effect of MeJA and MβCD on triterpenoid production in *C. reinhardtii* UVM4. Indeed, a synergistic effect on terpenoid production has previously been observed in plants. For instance, the combination of MeJA and MβCD significantly increased vindoline, catharanthine, and ajmalicine (monoterpenoid indole alkaloids) production in *Catharanthus roseus* cambial meristematic cell cultures compared to individual treatments [18,19]. The interactive effect of the two elicitors was also observed on taraxasterol and taraxerol (pentacyclic triterpenoids) production in *Taraxacum officinale* callus root culture [20]. A synergistic effect of MeJA and MβCD on the production of the antitumor diterpene alkaloid, taxol, was also reported in *Taxus x media* cells, reaching production levels 55 times higher than in non-elicited cultures, and 4 to 10 times higher than in cultures separately treated with MeJA and cyclodextrin, respectively [15]. Unlike in plants, we observed no synergistic effect of MeJA and MβCD in *C. reinhardtii* as the triterpene levels were not higher in cells treated with both elicitors than in cells treated with each elicitor individually.

## 2. Results

### 2.1. Methyl Jasmonate Triggers an Oxidative Stress in C. reinhardtii

**Figure 1:**
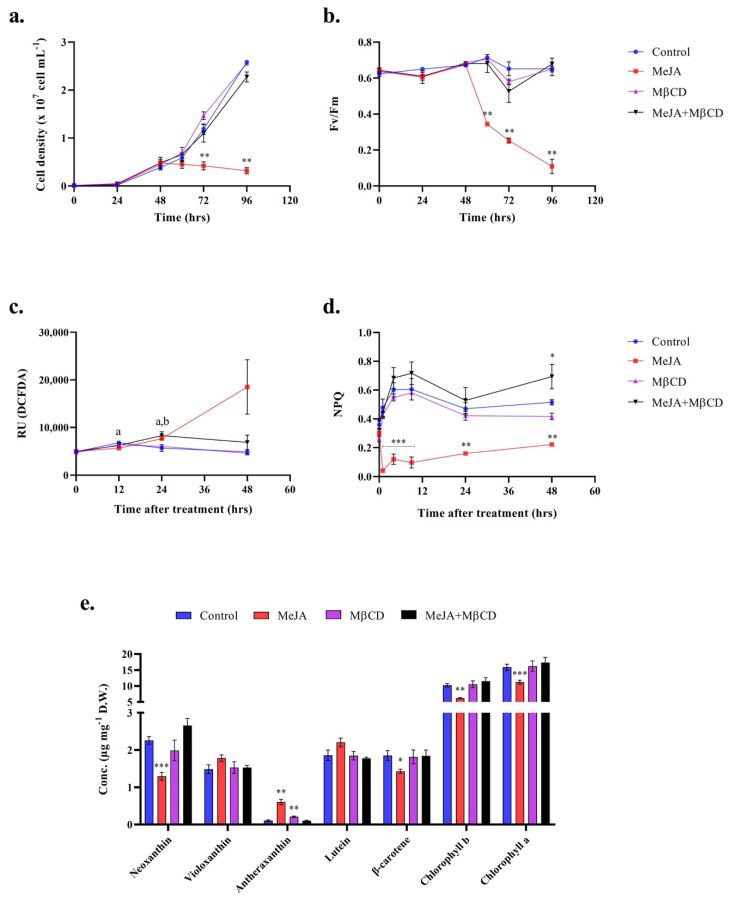
Monitoring of oxidative stress in *Chlamydomonas reinhardtii* UVM4 cells treated either with methyl jasmonate (MeJA, 1 mM), methyl-β-cyclodextrin (MβCD, 5 mM), or MeJA (1 mM) + MβCD (5 mM) compared to the control (1% ethanol). (**a**) Cell density and (**b**) photosynthetic activity throughout the experiment. The cells were treated after 48 h of growth, which is when (**c**) the reactive oxygen species (ROS) content of cells and (**d**) non-photochemical quenching (NPQ) after 10 min of high light, started to be monitored. For these measurements, the initial 0 h represents the time at which the cells were treated. The Relative Fluorescence Units of DCFDA dye was used to assess ROS content of cells. ^a^ indicates significant differences in ROS content between the control and MeJA treatment and ^b^ the difference between the control and MeJA + MβCD treatment (Dunnett’s, *p* < 0.05). (**e**) Pigments concentrations in *C. reinhardtii* UVM4 biomass after 48 h of treatment. Concentrations are reported in µg per mg of dried weight (D.W.). Significant differences between the control and the treatments are indicated as follows: * *p* < 0.05, ** *p* < 0.01, *** *p* < 0.001 (Dunnett’s). Mean ± SD is shown (*n* = 3).

*C. reinhardtii* UVM4 cultures were treated in early-exponential phase (48 h after inoculation) with either 1 mM of MeJA, 5 mM of MβCD or a combination of both. The addition of MeJA affected the cells physiology, unlike the other treatments, and resulted in significant reduction in cell growth and photosynthetic efficiency (Figure 1a,b). MeJA also triggered an oxidative stress as shown by the significant increase in reactive oxygen species (ROS) content in the cells (Figure 1c). The onset of oxidative stress did not result in higher cell mortality as the percentage of dead cells in the population remained under 0.25% until 48 h of treatment (Figure S1).

MβCD had no effect on the cell physiology. More surprisingly, when applied in combination with MeJA, it alleviated the effects of MeJA on the cell growth and photosynthetic activity (Figure 1a,b). A slight increase in ROS content was observed 24 h after treatment, but it decreased to control levels after 48 h (Figure 1c).

The increase in ROS was matched by a change in xanthophylls (i.e., noexanthin and antheraxanthin) abundance (Figure 1e). The xanthophyll zeaxanthin was not detected, as it is commonly present in very low quantity, thus making it hard to measure. Among all the treatments, only MeJA had an effect on pigment concentrations after 48 h, with the exception of a slight increase in antheraxanthin concentration in the presence of MβCD, which was not observed when MβCD was combined with MeJA (Figure 1e). The MeJA treatment also lead to lower chlorophylls and β-carotene contents. These results correlate with the decrease in the photosynthetic efficiency of photosystem II observed in Figure 1b. Impaired non-photochemical quenching (NPQ) in presence of MeJA also correlate with a damaged photosystem II, as NPQ is usually activated to dissipate excess photon energy as heat and to preserve the integrity of photosystem II (Figure 1d, Figure S2). MeJA affected *C. reinhardtii* cells immediately, as seen by impaired NPQ as early as 1 h after treatment, but NPQ seemed to recover over time. Interestingly, NPQ increased in the cells after 48 h of treatment with a combination of MeJA and MβCD.

### 2.2. MeJA and MβCD Have No Synergistic Effects on Triterpenoids Abundance

**Figure 2:**
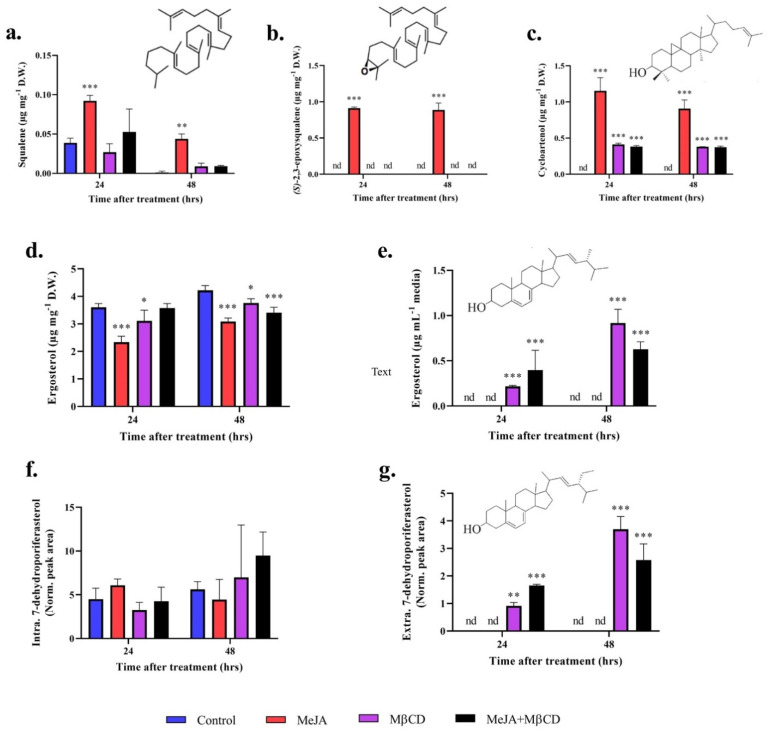
Triterpenoids concentrations in *C. reinhardtii* UVM4 biomass (**a**–**d**,**f**) and growth media (**e**,**g**) after 24 and 48 h of treatment with methyl jasmonate (MeJA, 1 mM), methyl-β-cyclodextrin (MβCD, 5 mM), or MeJA (1 mM) + MβCD (5 mM) compared to the control (1% ethanol). Intracellular concentrations of (**a**) squalene, (**b**) *(S)*-2,3-epoxysqualene, (**c**) cycloartenol, (**d**) ergosterol, and (**f**) 7-dehydroporiferasterol are reported in µg per mg of dried weight (D.W.). Extracellular concentrations of (**e**) ergosterol and (**g**) 7-dehydroporiferasterol are reported in µg per milliliter of growth media. Squalene, (S)-2,3-epoxysqualene and cycloartenol were not detected in the culture media. “nd” = not detected. Asterisks indicate significant differences between the control and the treatments (* *p* < 0.05, ** *p* < 0.01, *** *p* < 0.001, Dunnett’s). Mean ± SD is shown (*n* = 3).

Treatment with MeJA alone resulted in increased intracellular amount of squalene, (S)-2,3-epoxysqualene and cycloartenol from below detection levels in the control, up to 0.1, 1.0and 1.2 µg mg_D.W._^−1^, respectively (Figure 2a–c). Treatment with MβCD resulted in increased concentrations of cycloartenol only, which were 2-fold lower than the concentrations reported in presence of MeJA. Treatment with the mixture MeJA + MβCD followed the same trend as MβCD treatment alone and no statistical differences were detected between these two treatments (Figure 2a–c). No synergistic effect of MeJA and MβCD on triterpenoids abundance was observed. 

### 2.3. Cyclodextrin Sequesters Sterols to the Growth Media

The amount of cell-associated ergosterol was lower in the treatments than in the control, while the treatments had no effect on the 7-dehydroporiferasterol content (Figure 2d,f). The decrease in ergosterol content in the biomass treated with MeJA is in accordance with our previous study [13], but the underlying mechanism has yet to be elucidated. The cyclodextrin successfully exported both sterols (ergosterol and 7-dehydroporiferasterol) to the growth media (Figure 2e,g). For all the treatments, the sterols content increased overtime (Figure 2d–g). The sequestration of sterols to the growth media created a metabolic pull, which the cells tried to compensate by producing more sterols. Maintaining the sterol homeostasis steady is crucial for proper functioning. No synergistic effect of MeJA and MβCD on the abundance of the two main sterols was observed, as treatment with a combination of these two elicitors did not significantly affect the sterol content in the biomass or the medium compared to MβCD alone (Figure 2d–g). Squalene, (S)-2,3-epoxysqualene and cycloartenol were not detected in the culture media.

To exclude the possibility that the observed results were due to the absence of cell wall or mutations incurred by the strain UVM4, we subjected the *C. reinhardtii* strain 137c to the same treatments. Similar results were obtained (Figure S3), implying that the differential accumulation of triterpenoid intermediates and the lack of synergy is intrinsic to *C. reinhardtii*. 

## 3. Discussion

The need for cheaper and faster terpenes production is rising as new industrially relevant molecules are discovered. This paper presents a new biotechnological approach using two elicitors, MeJA and MβCD, to increase the production of *C. reinhardtii* valuable native terpenes precursors (e.g., squalene, *(S)*-2,3-epoxysqualene) suitable for the synthesis of more complex high-value triterpenes (e.g., betulinic acid [21], or semi-synthetic drugs [4]). Although, the use of MeJA and MβCD in large-scale cultures is not economically sustainable, this approach provides useful knowledge to understand mechanisms for improvements.

**Figure 3:**
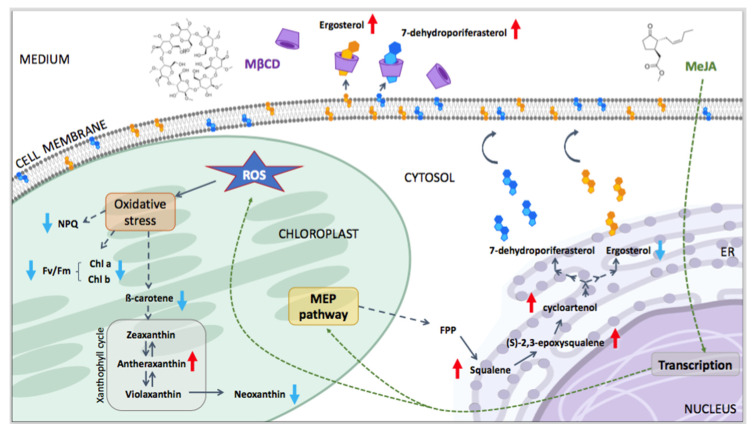
Conceptual diagram of the effects of methyl jasmonate (MeJA) and methyl-β-cyclodextrin (MβCD) on pigments and terpenes biosynthesis in *C. reinhardtii* UVM4, based on the results of this study. Ergosterol and 7-dehydroporiferasterol are the predominant sterols in the cell membrane of *C. reinhardtii* [3]. The cell wall is not shown as the strain UVM4 is cell-wall deficient. Triterpenes biosynthesis occurs in the endoplasmic reticulum (ER). In plants, MeJA triggers a transcriptional re-arrangement in the nucleus, which then starts a coordinated transcriptional response leading to an up-regulation of the MEP pathway and an oxidative stress. A similar response has been observed in *C. reinhardtii*, although it lacks orthologs of key genes involved in MeJA signaling in plants [13]. The red and blue arrows indicate an increase and decrease in metabolites abundance compared to untreated cells, respectively. Dashed arrows indicate multiple enzymatic steps. MEP: 2-C-methyl-D-erythritol 4-phosphate; ROS: reactive oxygen species; Chl: chlorophyll; FPP: farnesyl diphosphate.

The results presented in this study correlate with the observations of our previous study investigating the effect of MeJA on the MEP pathway and terpenes biosynthesis in the wild-type strain *C. reinhardtii* 137c [13]. A decrease in chlorophylls, β-carotene, and sterols coupled with an increase in squalene and *(S)*-2,3-epoxysqualene was reported [13]. The present study brings new elements such as an increase in ROS and the photoprotective pigment antheraxanthin, and an increase in cycloartenol production coupled with export of the dominant sterols into the growth medium in the presence of MβCD (Figure 3). These results were reported for the strain UVM4, optimised for genetic engineering; however, strain 137c is responding in a similar way in the presence of the two elicitors (Figure S3). 

Application of MeJA caused a senescence-like symptom in *C. reinhardtii* cells as indicated by a great decline in chlorophylls, photosynthesis activity and growth. The accumulation of ROS and antheraxanthin implies that an oxidative stress is probably instigating this symptom. MeJA is known to trigger an oxidative stress in plants by inducing the production of ROS first in the mitochondria and subsequently in the chloroplasts [14,22]. Oxidative stress is one of the main causes of MEP pathway re-arrangement and up-regulation in plant cells, as certain products of this pathway (including carotenoids, tocopherols and isoprene) are involved in protection against oxidative stress [23]. Our previous study showed that MeJA up-regulated the MEP pathway in *C. reinhardtii*; however, this current work brings evidence that it is doing so by prompting an oxidative stress. Together the results are providing the first evidence that, like in plants, the MEP pathway of green algae also responds to oxidative stress. Nonetheless, *C. reinhardtii* lacks orthologs of key genes involved in MeJA signaling in plants and future studies should focus on solving the mystery of MeJA signal transduction in *C. reinhardtii*.

Like in *C. reinhardtii*, the addition of exogenous MeJA in plants lead to losses of chlorophylls, β-carotene and lutein, while the xanthophylls, antheraxanthin and zeaxanthin, accumulate and the photosynthesis activity reduces [24,25]. Antheraxanthin and zeaxanthin are photoprotective pigments produced from the conversion of violaxanthin in the xanthophyll cycle (Figure 3) by the enzyme violaxanthin de-epoxidase (VDE). In plants, these pigments accumulate in presence of the ROS scavenger, ascorbic acid, to promote non-photochemical quenching (NPQ) under high light stress [26,27]. While the contribution of antheraxanthin and zeaxanthin to NPQ is constitutive in higher plants, it was reported to be only minor, if any, in *C. reinhardtii* [28,29]. Our data corroborate these observations, as the accumulation of antheraxanthin was coupled with a decrease in NPQ and not an increase as reported in plants. MeJA significantly impaired NPQ in *C. reinhardtii* through an unclear mechanism, likely linked to a direct or indirect effect of increased ROS. The main component of NPQ is energy quenching, qE, which in green algae depends on the Light-Harvesting Complex Stress-Related (LHCSR) proteins [30]. The quenching function of LHCSR depends on its binding to pigments such as chlorophylls and xanthophylls [30]. The lower abundance of some of these pigments during MeJA treatment, could have affected the LHCSR function leading to impaired NPQ. A similar response was observed in a qE-deficient mutant of *C. reinhardtii*, npq4, lacking two of the three genes encoding LHCSR proteins [28]. It is possible that MeJA affects the production of LHCSR proteins in *C. reinhardtii*. However, the abundance of these proteins was not investigated in this work but should be considered for further studies to provide more conclusive results. *C. reinhardtii* possesses an atypical VDE, which is not homologous to the plant VDE and does not require the presence of ascorbate [31,32]. However, the current study shows that, like in plants, MeJA treatment activates VDE and induces antheraxanthin and probably zeaxanthin accumulation in *C. reinhardtii*. The carbon flux being forced towards the accumulation of antheraxanthin, a concomitant reduction in carotenoids upstream or downstream of the xanthophyll cycle (β-carotene and neoxanthin, respectively) was observed (Figure 3). In general, *C. reinhardtii* seems to respond to MeJA treatment in a very similar way to higher plants. To better understand the mechanism of protection of *C. reinhardtii* towards MeJA-induced oxidative stress, further studies could investigate the role of enzymatic antioxidants such as catalase, peroxidase, superoxide dismutase and glutathione reductase, the activities of which are known to increase greatly in MeJA-treated plants [24]. 

The absence of synergy between MβCD and MeJA in *C. reinhardtii* UVM4 was unexpected. Indeed, we hypothesised that the concentrations of terpenes would increase even further in the presence of the two elicitors, especially in the case of cycloartenol as its concentration increased in separate treatments with MeJA and MβCD when compared to non-elicited cells (Figure 2c), but this is not what we observed. Instead, MβCD seemed to counteract the effect of MeJA as no significant differences were observed between the MβCD alone and MβCD + MeJA treatments for all the performed analyses. Similarly, Briceño et al. [33] showed no synergistic effect of MeJA and MβCD on the extracellular concentration of phytosterols (isofucosterol and β-sitosterol) and taraxasterol in Micro-Tom tomato cell cultures. However, a synergistic effect on terpenoids production has previously been observed in plants [15,18,19,20]. For instance, Sabater-Jara et al. reported a drastic increase in taxol when *Taxus x media* cells were treated with both elicitors compare to cultures treated independently [15]. Interestingly, the authors also noticed that the negative effect of MeJA on *Taxus* cell growth and viability was reduced when the cells were previously treated with cyclodextrins [15]. A similar phenomenon was observed in the current study, where MβCD offset the negative effect of MeJA on *C. reinhardtii* UVM4 growth and photosynthetic efficiency when added simultaneously. MβCD has been shown to increase MeJA solubility in water [34], hence it could be anticipated that MβCD would exacerbate the MeJA effect instead of offsetting it. However, this is not what we observed when treating the cells with the two elicitors simultaneously. Different results might have been obtained if the cells were initially treated with MeJA before adding MβCD, but it remains to be proven. The mechanism behind the neutralisation of MeJA effects by MβCD is yet to be deciphered as no data on algal cell membrane permeability, cross reactions or complex formation has been reported in the literature. The depletion of sterols in the cell membrane following MβCD treatment could alter the perception of MeJA signal in *C. reinhardtii* in a similar way that it affects plant membrane receptors. For instance, the disruption of sterols by MβCD treatment has been shown to significantly affect the innate immunity of *Arabidopsis* by altering the plant transmembrane receptor kinase FLAGELLIN SENSING 2 (FLS2) [35]. However, the MeJA signaling machinery in *C. reinhardtii* being distinct from higher plants and unknown [13], the assumption that a sterol-dependent receptor is involved in MeJA signaling in *C. reinhardtii* will have to be investigated further.

The addition of MeJA affected triterpenoids production higher up in the pathway, while MβCD acted only on the later products of the sterol pathway (Figure 3). MeJA has been shown to up-regulate the 2-C-methyl-D-erythritol 4-phosphate (MEP) pathway and triterpenoid biosynthesis up to cycloartenol in *C. reinhardtii* [13]. Similar results were obtained in this study, where MeJA elicited the production of squalene, *(S)*-2,3-epoxysqualene and cycloartenol, but it reduced the abundance of ergosterol in the biomass and had no effect on the extracellular sterols content (Figure 2). On the other hand, MβCD had no effect on the biosynthesis of squalene and *(S)*-2,3-epoxysqualene, but it triggered the accumulation of cycloartenol (to a lesser extent than MeJA), a slight decrease in intracellular ergosterol, and a drastic increase in extracellular sterols content (ergosterol and 7-dehydroporiferasterol). The total amount of sterols increased overtime, probably because the excretion of these molecules altered the final sterol profile of the cell, which is usually kept under strict control, therefore forcing the cell to produce more sterols to maintain sterol homeostasis [36,37]. Similarly, Miras-Moreno et al. [38] observed no effect of MβCD on the expression of genes coding for the squalene synthase (SQS) and the cycloartenol synthase (CAS) in cyclodextrin-treated carrot cell suspension but reported an accumulation of phytosterols in the extracellular medium. In the current study, the addition of MβCD affected the triterpenoid biosynthesis from cycloartenol, while MeJA impacted triterpenoids production from the production of precursors in the MEP pathway to the final sterol products. This is the first time MβCD and the MeJA + MβCD combination were tested in microalgae. The absence of synergy between the two elicitors suggests that using them in combination is not a suitable strategy to boost triterpenoid biosynthesis in *C. reinhardtii* UVM4. However, MeJA has proven to be the best elicitor for the production of soluble high-value triterpenoids, while MβCD successfully exported membrane-bound sterols to the growth media. The choice of the elicitor depends on the desired product and its localisation within the cell. The ability to sequester targeted products into the culture medium is important from a biotechnological point of view. It allows continuous production without the need to harvest the cells and simplify the downstream purification process, inevitably reducing the overall production cost.

## 4. Materials and Methods

### 4.1. Strains, Culture Conditions and Treatments

The strain *Chlamydomonas reinhardtii* UVM4 was graciously provided by Prof. Ralph Bock and *C. reinhardtii* wild type strain 137c (also named strain CC-125 mt+) was purchased from ThermoFisher Scientific (North Ryde, Australia) as part of the Invitrogen GeneArt^®^ Chlamydomonas Protein Expression Kit. Both strains were cultivated in 1 L glass Erlenmeyer flasks in mixotrophic conditions in Tris-acetate-phosphate (TAP) medium at 25 °C under 50 ± 10 μmol photon m^−2^ s^−1^ (continuous illumination) with constant agitation at 100 rpm. Continuous illumination was chosen over light–dark cycles to boost the growth and maximise the biomass. The cultures were inoculated with a stationary phase culture at a 1:50 inoculum to medium ratio. The growth was monitored by spectrophotometry at OD_750nm_ (UV-1280, Shimadzu) and converted to cell density using the following equation: Cell density = (OD_750nm_−0.088)/9 × 10^−8^ (as per GeneArt^®^ Chlamydomonas Protein Expression Kit user manual). The cells were treated in early log phase (cell density of~5 × 10^6^ cells mL^−1^), 48 h after inoculation, with MeJA diluted in 100% ethanol to a final concentration of 1 mM (1% ethanol), and MβCD (final concentration of 5mM) diluted in TAP medium and filtered sterilised before addition. The control was treated with 1% ethanol. The cells were treated again with MβCD (5 mM) 24 h later. TAP medium supplemented with MeJA and/or ethanol was also added to the corresponding flasks 24 h later, to maintain a constant volume of medium in all the treatments. 

### 4.2. Photosynthetic Activity and NPQ

The maximum quantum yield (Fv/Fm) of photosystem II was used as a proxy for *C. reinhardtii* photosynthetic activity during MeJA and MβCD treatments. Fv/Fm was measured by pulse-amplitude modulated fluorometry (PAM) using a POCKET-PAM (Gademann Instruments GmbH, Würzburg, Germany) after at least 10 min of dark acclimation. The measurements were performed at room temperature from the bottom on the glass Erlenmeyer flask, with the following settings: blue light, measuring light intensity < 0.2 µmol photons m^−2^ s^−1^ PAR, saturation pulse intensity of 1200 μmol photons m^−2^ s^−1^ PAR, and saturation pulse width of 1 s.

Non-photochemical quenching (NPQ) was measured using an Imaging PAM (Heinz Walz GmbH, Effeltrich, Germany). A total of 200 µL of each sample were transferred to a 96 well plate (Bio-Rad Laboratories Pty Ltd, Gladesville, Australia, catalogue number HSP9601), dark adapted for 10 min and immediately exposed to a saturation light pulse of blue actinic light. NPQ was determined after 10 min of exposure to high light (800 µmol photons m^−2^ s^−1^). 

### 4.3. ROS Detection

The cells were harvested at 4000 × *g* for 5 min and resuspended in TAP medium to a density of 1 × 10^6^ cells mL^−1^. The samples were stained with DCFDA at a final concentration of 20 µM (Cellular ROS Assay kit, Abcam Australia Pty Ltd, Melbourne) for 30 min at 37 °C and then left at room temperature for another 2 h before being processed on a flow cytometer (CytoFLEX S, Beckman Coulter, Lane Cove West, Australia). Twenty thousand cells were analysed for detection of DCFDA fluorescence, excited by 488 nm laser and detected in a 525 ± 40 nm channel. Cells treated with 1 mM tert-butyl Hydrogen Peroxide (tbHP) were used as positive control to set DCFDA positive gate. The stock solution of DCFDA dye was diluted to 2 mM in DMSO (final concentration of 1% DMSO in the samples) based on preliminary results showing that *C. reinhardtii* cells can tolerate up to 1% DMSO without affecting the assay (data not shown). 

### 4.4. Viability Analysis

Samples containing ~1 × 10^6^ cells mL^−1^ were processed as per supplier protocol (LIVE/DEAD^TM^ fixable Violet Dead Cell Stain kit, ThermoFisher Scientific, North Ryde Australia). Briefly, cells were washed with phosphate buffered saline (PBS) 1×, stained 30 min at RT in the dark with violet fluorescent reactive dye diluted in DMSO, fixed in 1.6% paraformaldehyde solution in PBS 1x and stored at 4 °C for up to 5 days. The samples were then rinsed in PBS 1x supplemented with 1% bovine serum albumin (BSA) and 30,000 cells were processed on a flow cytometer (CytoFLEX S, Beckman Coulter) with 488 nm excitation and 525 ± 40 nm emission. The gating was performed based on a control prepared by mixing 50:50 “live” and “dead” (boiled for 3 min in a water bath) *C. reinhardtii* cells, which were processed at the same time and in the same conditions as the samples.

### 4.5. Metabolites Extraction and Quantification

Algal cultures were harvested (25 and 20 mL for triterpenoids and pigments analyses, respectively) at 24 and 48 h after treatment. The cells were pelleted at 4000 × *g* for 5 min, rinsed once with TAP medium, snap froze and stored at −20 °C. Cells were freeze-dried and the dry weights were recorded. Extractions and analyses by gas chromatography–mass spectrometry (GC–MS) for triterpenoids and HPLC-UV for pigments were performed as previously described in [13]. 

## 5. Conclusions

This study showed that MeJA triggers an oxidative stress in *C. reinhardtii*. This observation together with our previous work provides the first evidence that the MEP pathway and the triterpenoid pathway(s) of green algae respond to oxidative stress. This finding is likely to spark new research to understand the underlying molecular mechanisms. The first strategy for active secretion of triterpenoids was reported in green algae, which is very useful for future biotechnological applications, such as the production and purification of high-value plant triterpenoids in *C. reinhardtii*. The exogenous addition of MβCD created a metabolic pull towards the production of sterols. This approach can be used to up-regulate triterpene pathways in *C. reinhardtii*. Adequate strategies to enrich the microalgal biomass with triterpenoids precursors and intermediates will pave the way for efficient genetic engineering in these organisms.

## Data Availability

Data is contained within the article or supplementary material. The raw data are available on request from the corresponding author.

## Supplementary Materials

The following are available online at https://www.mdpi.com/1424-8247/14/2/125/s1, Figure S1: Percentage of dead cells in the population, Figure S2: Non-photochemical quenching measurements, Figure S3: Triterpenoids concentrations in wild-type *C. reinhardtii* 137c biomass (a) and growth media (b) after 48 h of treatment.
