# Supplementary material for: Methyl Jasmonate and Methyl-β-Cyclodextrin Individually Boost Triterpenoid Biosynthesis in Chlamydomonas Reinhardtii UVM4
Source: Pharmaceuticals (Basel). 2021 Feb 5;14(2):125. doi: 10.3390/ph14020125 (PMC7915139; doi:10.3390/ph14020125)
Supplement: Supplementary file 1 [file pharmaceuticals-14-00125-s001.pdf]

pharmaceuticals

Supplementary Material

Methyl jasmonate and methyl-β-cyclodextrin individually boost triterpenoid biosynthesis in *Chlamydomonas reinhardtii* UVM4

Audrey S. Commault [1,*], Unnikrishnan Kuzhiumparambil [1], Andrei Herdean [1], Michele Fabris [1,2], Ana Cristina Jaramillo-Madrid [1], Raffaela M. Abbriano [1], Peter J. Ralph [1], Mathieu Pernice [1].

[1] University of Technology Sydney, Climate Change Cluster, Ultimo, NSW 2007, Australia
[2] CSIRO, Synthetic Biology Future Science Platform, Brisbane, QLD 4001, Australia;
unnikrishnan.kuzhiumparambil@uts.edu.au; andrei.herdean@uts.edu.au; michele.fabris@uts.edu.au; anacristina.jaramillomadrid@alumni.uts.edu.au; raffaela.abbriano@uts.edu.au; peter.ralph@uts.edu.au; mathieu.pernice@uts.edu.au
* Correspondence: audrey.commault@gmail.com

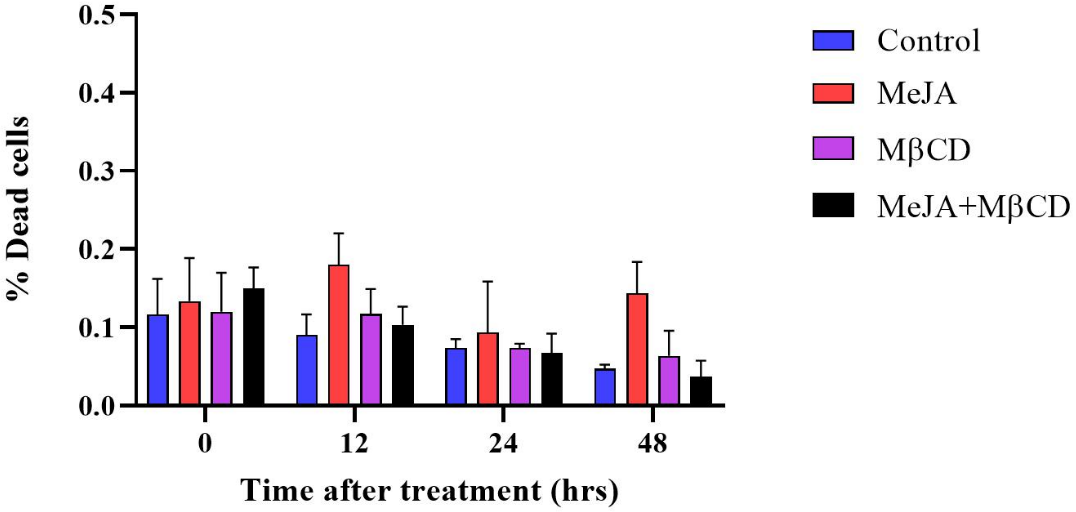

Figure S1. Percentage of dead cells in the population. No significant differences observed between the control and the treatments (Dunnett's, $p > .05$). Mean ± SD is shown (n = 3).

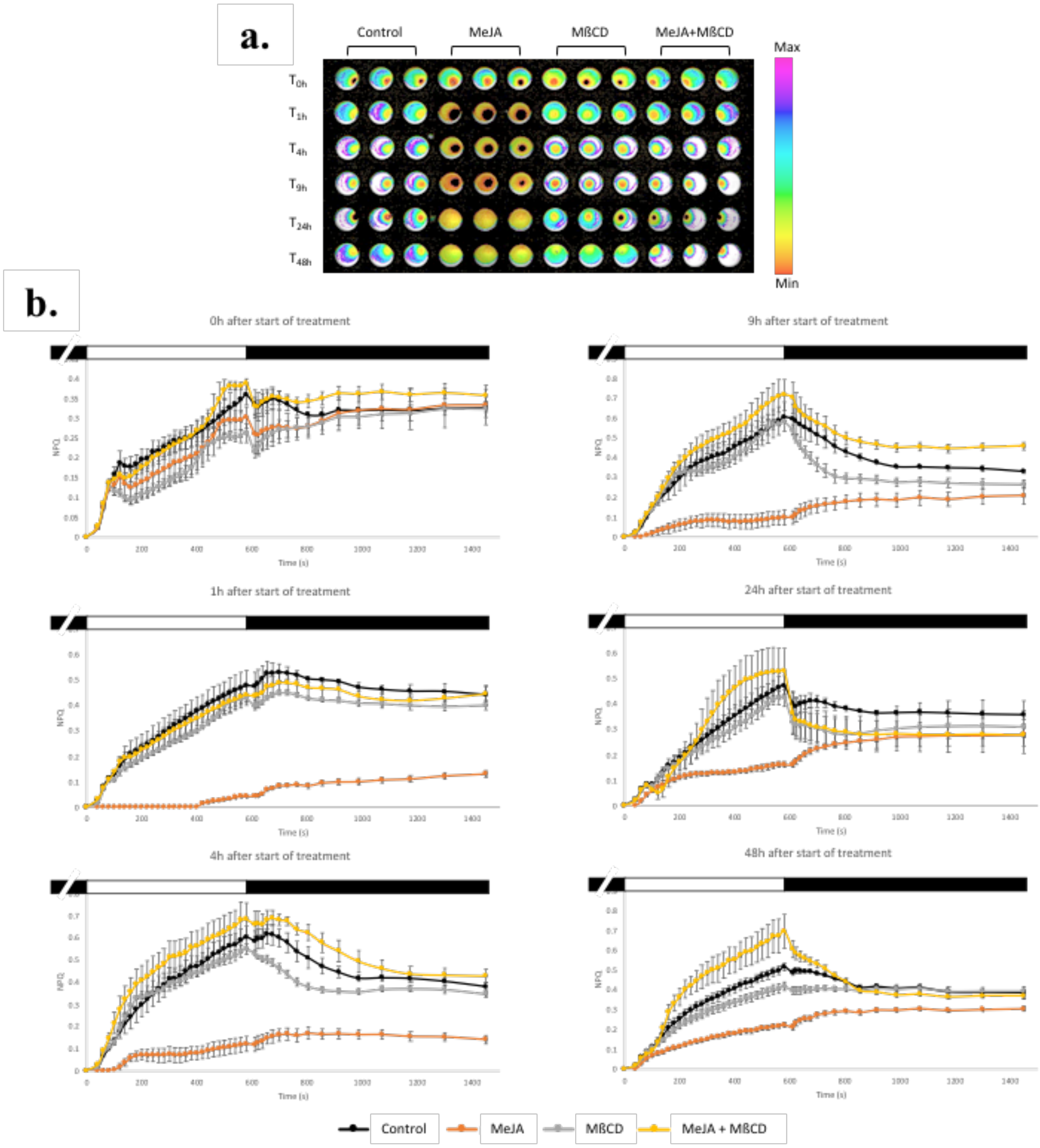

Figure S2. Non-photochemical quenching measurements. (a) Picture of the well plate used to measure NPQ after 10 minutes of exposure to high light. Colors indicate calculated NPQ. (b) NPQ induction and relaxation kinetics at 0h, 1h, 4h, 9h, 24h and 48h after treatment with methyl-jasmonate (MeJA, 1mM), methyl-β-cyclodextrin (MβCD, 5 mM) and a combination of both (MeJA + MβCD). The white and black bars above the graphs represent the periods of illumination and darkness, respectively. The light was turned off after 10 min. Mean ± SD is shown (n = 3).

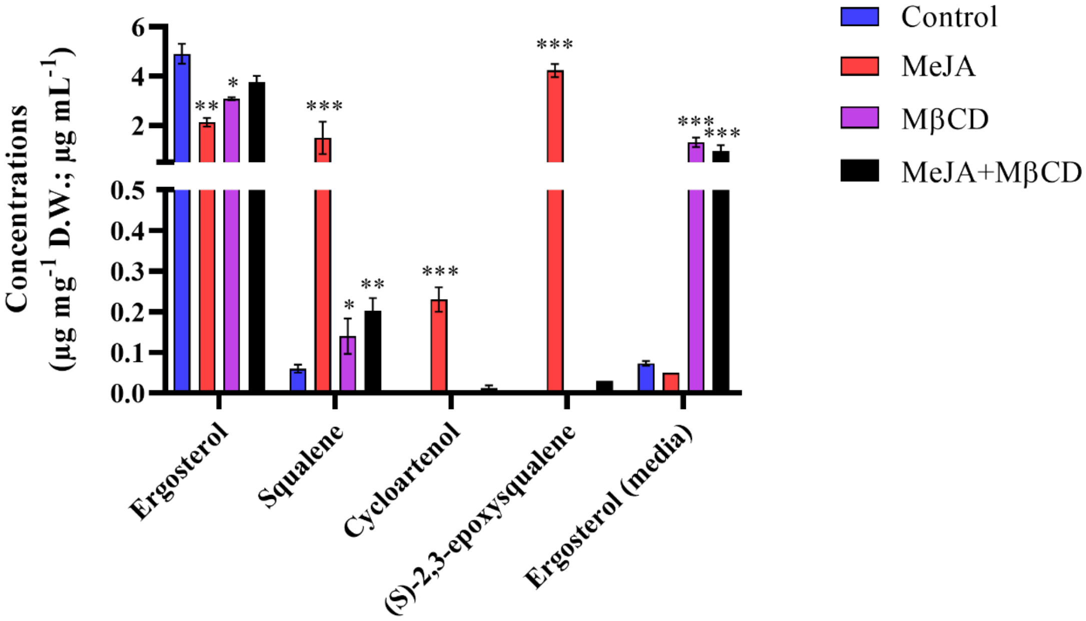

Figure S3. Triterpenoids concentrations in wild-type *C. reinhardtii* 137c biomass and growth media after 48 hours of treatment with methyl-jasmonate (MeJA, 1mM), methyl-β-cyclodextrin (MβCD, 5 mM) and a combination of both (MeJA + MβCD). Intracellular concentrations are reported in µg per mg of dried weight (D.W.), while extracellular concentrations are reported in µg per milliliter of growth media. “nd” = not detected. Asterisks indicate significant differences between the control and the treatments (** $p < .01$, *** $p < .001$, Dunnett's). Mean ± SD is shown (n = 3).
